# Chaperonin containing TCP-1 subunit 3 is critical for gastric cancer growth

**DOI:** 10.18632/oncotarget.22838

**Published:** 2017-12-01

**Authors:** Li-Juan Li, Lian-Sheng Zhang, Zhi-Jian Han, Zhi-Yun He, Hao Chen, Yu-Min Li

**Affiliations:** ^1^ School of Life Sciences, Lanzhou University, Lanzhou 730030, P.R. China; ^2^ Department of Hematology, Gansu Provincial Key Laboratory of Hematology, Second Hospital of Lanzhou University, Lanzhou 730030, P.R. China; ^3^ Department of General Surgery, Gansu Provincial Key Laboratory of Digestive System Tumors, Second Hospital of Lanzhou University, Lanzhou 730030, P.R. China

**Keywords:** gastric cancer, chaperonin containing TCP-1, CCT3, TRiC

## Abstract

**Background:**

Members of eukaryotic chaperonin family are essential for cell survival. Dysregulation of Chaperonin containing TCP-1 subunit 3 (CCT3) has been implicated in the development of several types of cancers. However, the role of CCT3 in the development of gastric cancer has yet to be determined.

**Methods:**

The expression patterns of CCT3 in the surgical specimens from 26 gastric cancer patients were evaluated using immunohistochemistry methods. To study the possible roles of CCT3 in the growth and survival of gastric cancer cells, RNA interference was used to knockdown CCT3 expression in gastric cancer cell lines BGC-823 and MGC-803. The effects of CCT3 knockdown on cancer cell proliferation, apoptosis and *in vivo* growth were examined. Finally, gene expression changes related to CCT3 knockdown were studied using gene array analysis and western blotting.

**Results:**

Higher level of CCT3 expression was detected in the gastric cancer tissue compared to adjacent non-cancerous epithelium. Knockdown of CCT3 inhibited proliferation and colony formation while promoted apoptosis of gastric cancer cells *in vitro*. Gastric cancer cells exhibited lower growth potential in nude mice when CCT3 expression was suppressed. Gene expression analysis showed that CCT3 knockdown was associated with down-regulation of mitogen-activated protein kinase kinase kinase 7, cell division cycle 42, cyclin D3 and up-regulation of cyclin-dependent kinase 2 and 6.

**Conclusion:**

Our results suggested that CCT3 played a critical role in gastric cancer growth and survival. Further studies on the mechanisms of CCT3 function is mandated to develop novel cancer treatment targeting CCT3.

## INTRODUCTION

Gastric cancer is the fourth most common malignancy worldwide and the second most common and lethal cancer in China. It was estimated that gastric cancer caused 498,000 deaths in China in 2015 [1]. Risk factors for gastric cancer, such as H. pylori infection, smoking and dietary toxin have been well established, allowing for implementation of primary prevention strategies. However, molecular mechanisms for gastric cancer development remain largely unknown. The identification of key molecules and pathways in gastric cancer is a critical step to the cure of the deadly disease. The molecular chaperone network (including chaperons and chaperonins) plays a central role in maintaining protein homeostasis (proteostasis) and proteome integrity. In the cytosol, a diverse group of chaperones cooperate in evolutionarily conserved pathways to guide the folding, intracellular localization, and proteolytic turnover of proteins [2]. The primary functions of molecular chaperones rely on their ability to transiently bind to hydrophobic regions of nascent or stress-denatured polypeptides and prevent misfolding or aggregation of the proteins. In addition to their pivotal role in ensuring proper folding of polypeptides, molecular chaperones play active role in protein degradation by maintaining target proteins in unfolded state [3]. For aggregated proteins that cannot be unfolded, chaperone-mediated autophagy or chaperone-assisted selective autophagy pathways are required for removal of the damaged proteins [4–6]. Proteostasis is vital for normal cellular functions and thus, disrupted proteostasis underlies various diseases and conditions including cancers. Inevitably, members of molecular chaperone pathway have been implicated in the development of cancers [7–9].

Chaperonin containing TCP-1 (CCT, also known as TRiC) belongs to group II (eukaryotic) chaperonin family, which is estimated to interact with 5-10% of proteome and is essential for cell survival [10]. CCT features a cylindrical architecture composed of two rings stacked opposite one another. The central cavity of CCT encapsulate substrate protein molecule, one at a time, allowing for proper folding without aggregation. Each ring is composed of 8 homologous but distinct subunits (CCT1-8), which recognize different motif within substrate proteins. The specific arrangement of these subunits provides the ability to fold certain proteins that cannot be folded by simpler heat shock protein family.

CCT has been shown to mediate the folding of a number of proteins implicated in oncogenesis such as tumor suppressor Von Hippel-Lindau (VHL), p53, pro-oncogenic proteins signal transducer and activator of transcription 3 (STAT3), and cell cycle regulatory proteins cell division cycle protein 20 (CDC20) [11–14]. Evidence is now emerging that CCT and its subunits are critical for the development of breast cancer and acute myeloid leukemia [15, 16]. More recent studies have shown CCT subunit 3 (CCT3) upregulation in hepatocellular carcinoma (HCC), cholangiocarcinoma and colon cancer [17–19], and that overexpression of CCT3 in HCC patients is associated with poor prognosis [20].

The involvement of CCT3 in gastric cancer remains unknown. In current study, expression of CCT3 in gastric cancer tissue was probed and the possible role of CCT3 in gastric cancer was explored in cell culture and xenograft animal models.

## RESULTS

### CCT3 was upregulated in gastric cancer tissue

**Figure 1:**
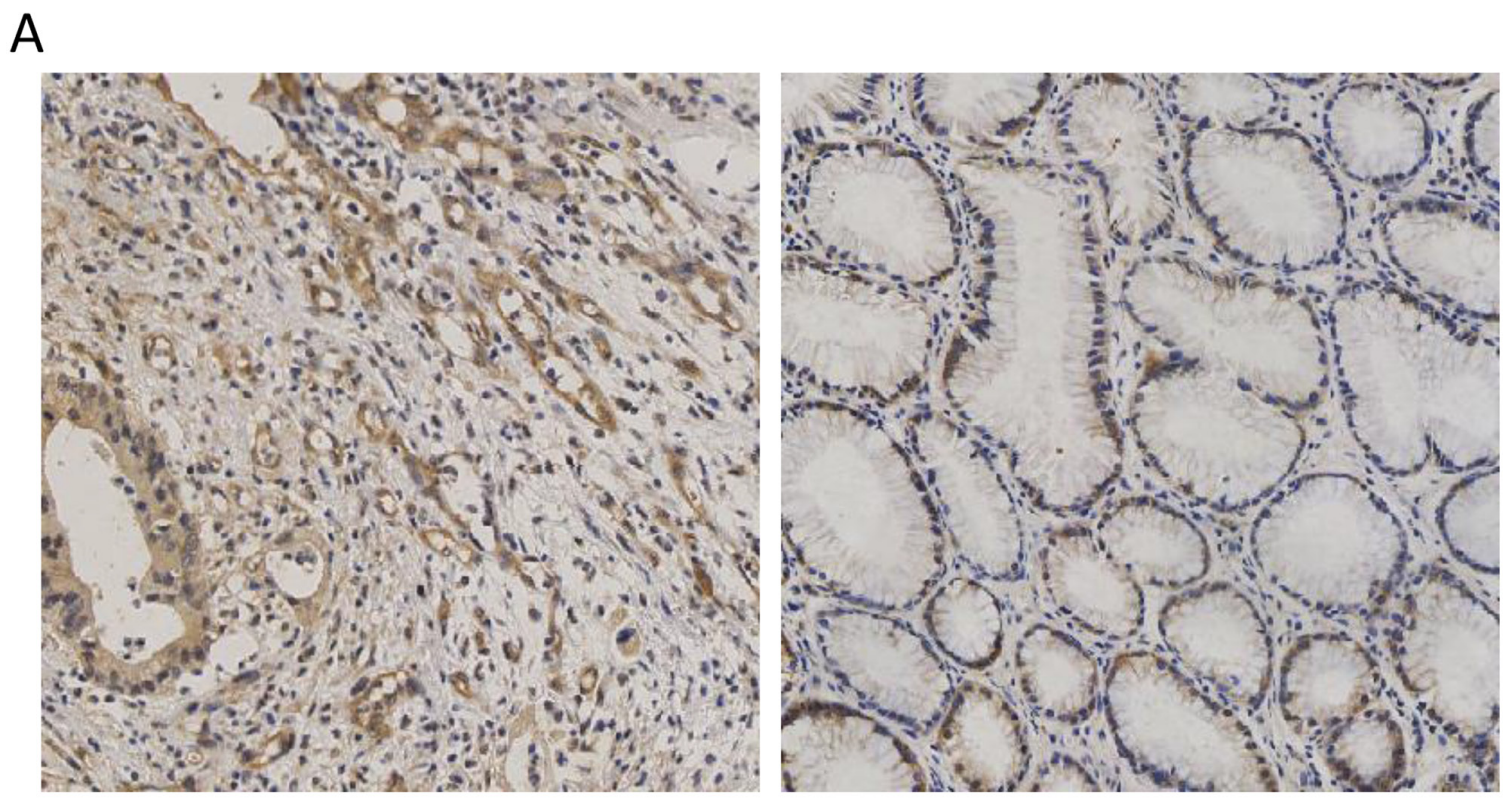
Expression of CCT3 in the gastric cancer and non-cancerous gastric epithelium **(A)** Higher level of CCT3 (Brown) was expressed in gastric cancer cells. **(B)** Lower CCT3 expression was seen in adjacent non-cancerous epithelium.

To examine the expression of CCT3 in gastric cancer, surgical samples from 26 gastric cancer patients were collected for immunohistochemistry staining. Sections of primary tumor and adjacent non-cancerous tissue from same patient were evaluated as matched pairs. Four primary tumor sections and four adjacent tissue sections from each patient were evaluated. Cytosolic CCT3 expression was higher in cancer cells compared to adjacent non-cancerous epithelium (Figure 1; Table 1, P<0.001, Fisher’s exact test)

**Table 1:** Expression of CCT3 in gastric cancer and non-cancerous tissue

	Low CCT3 expression	High CCT3 expression
Primary tumor	57	47
Adjacent non-cancerous epithelium	103	1

### Knockdown of CCT3 expression inhibited cancer cell growth *in vitro*

**Figure 2:**
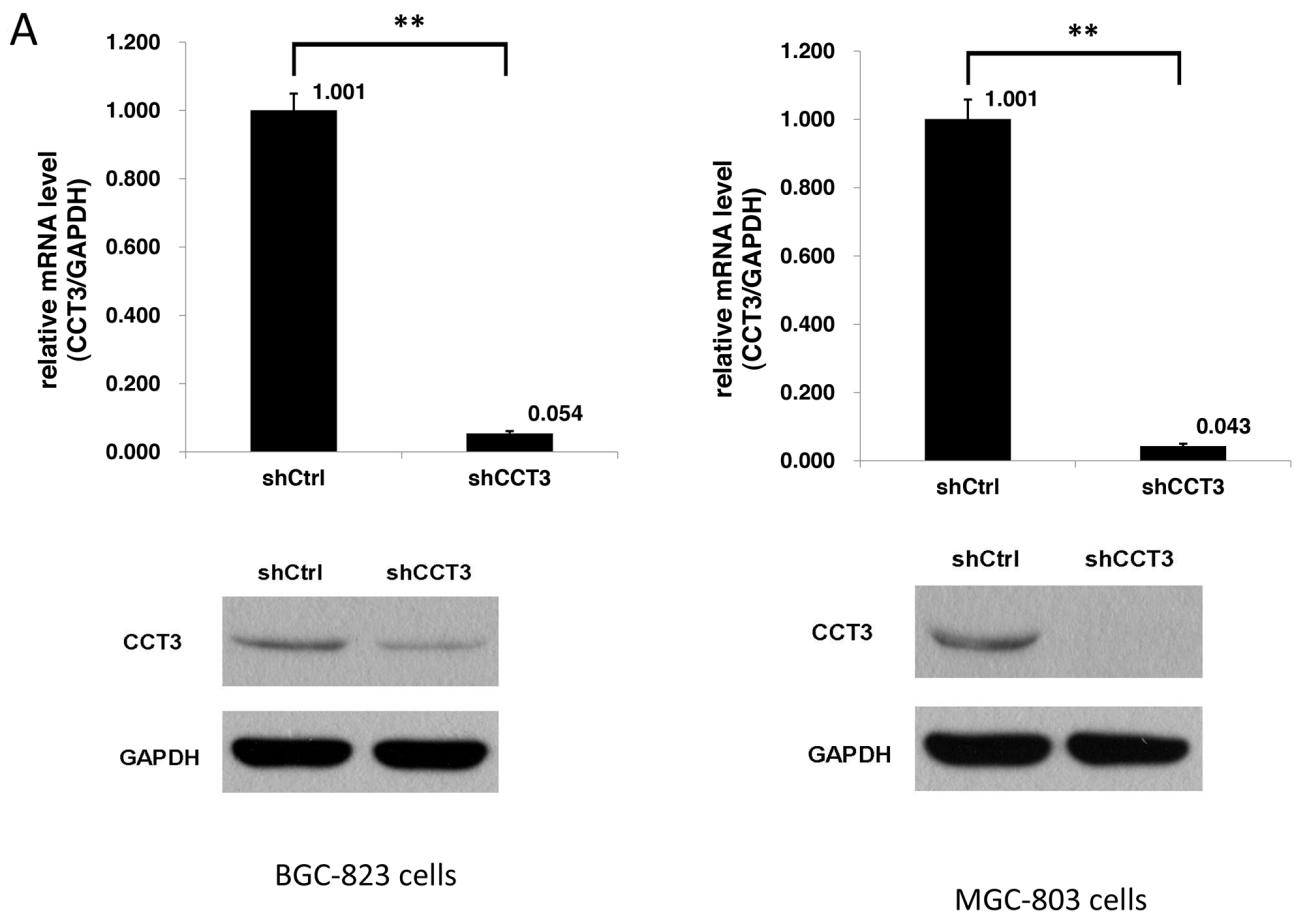

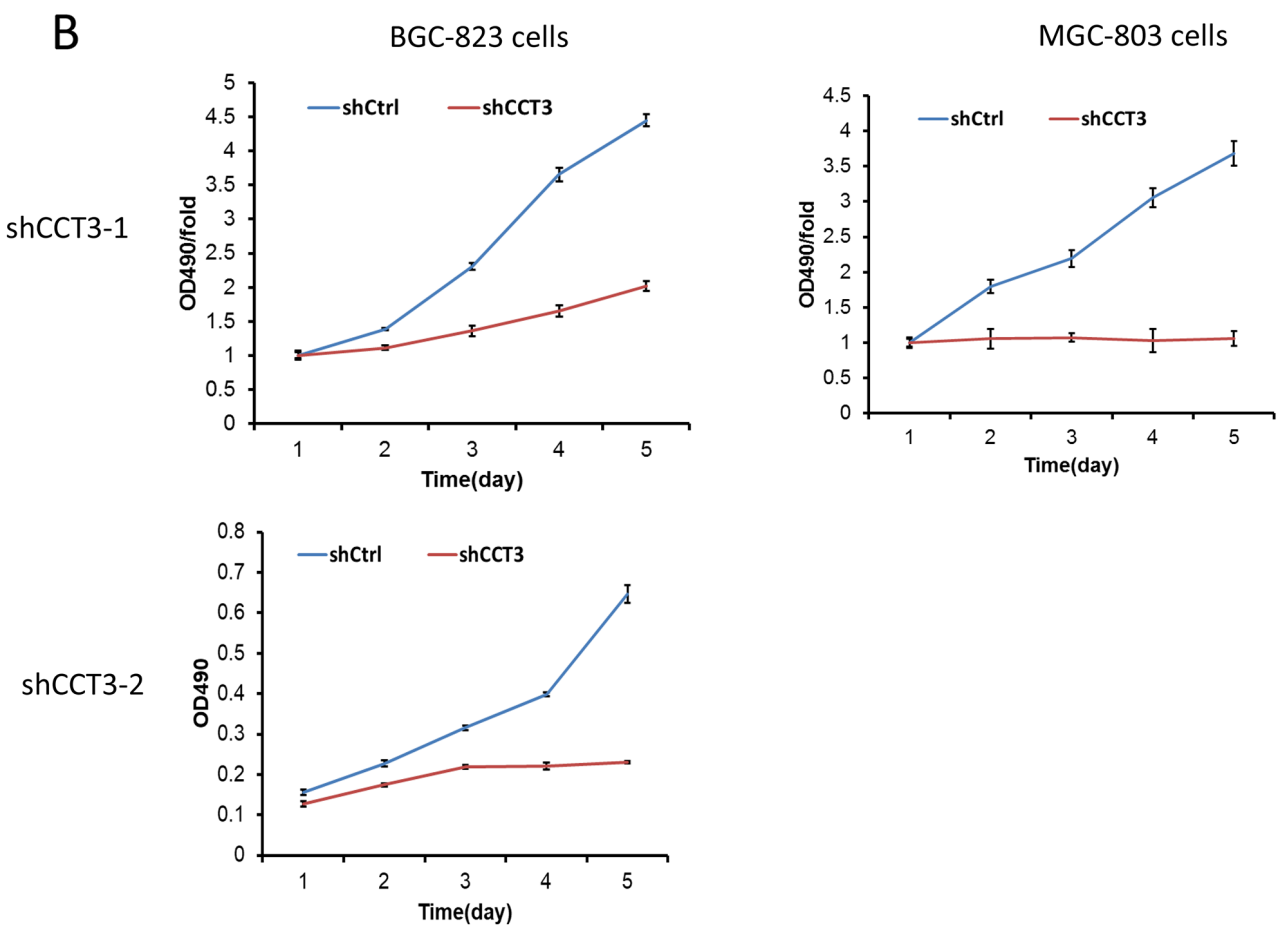

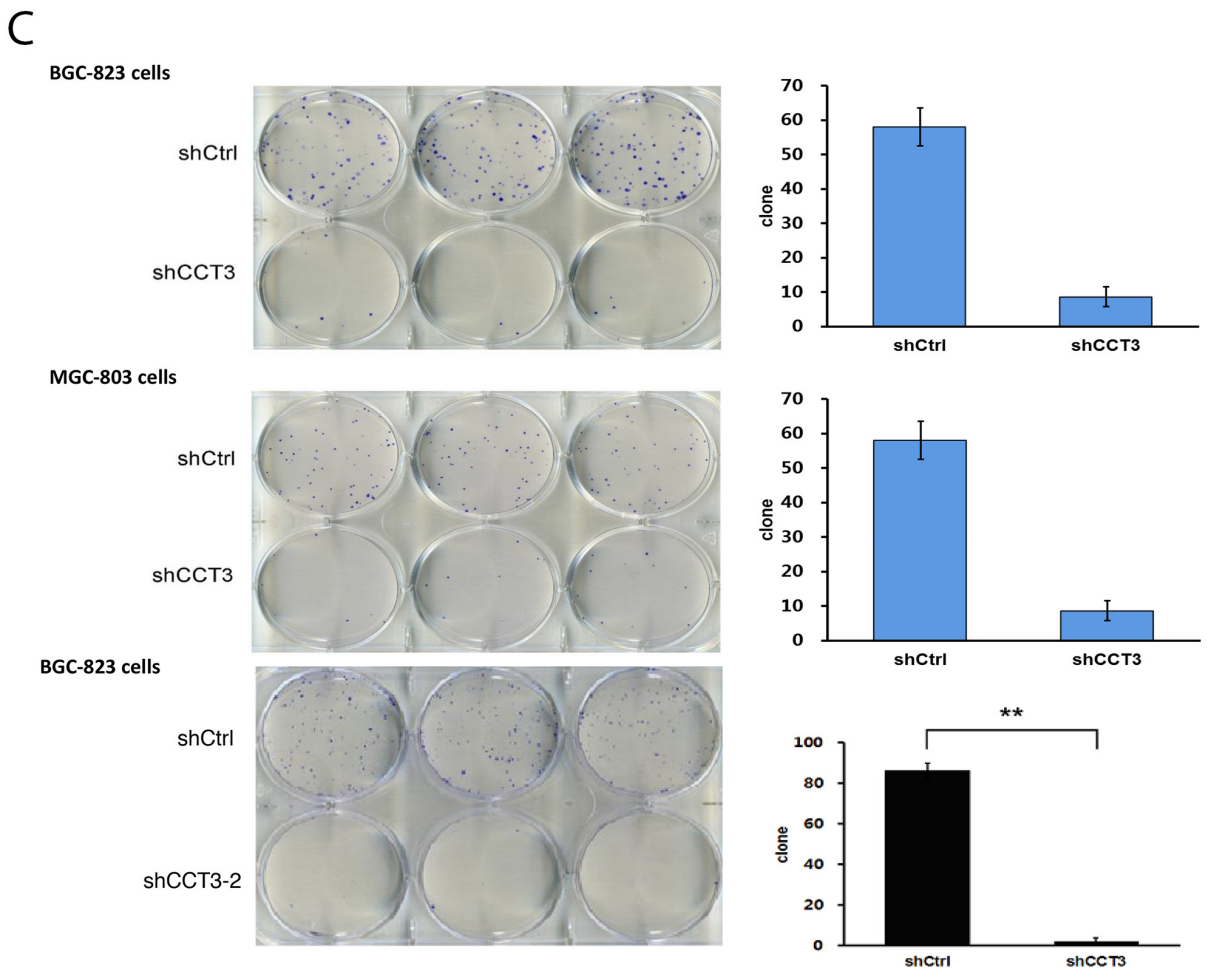
Knockdown of CCT3 inhibited gastric cancer cell growth **(A)** Knockdown of CCT3 in gastric cancer cell lines. After shCCT3 lentivirus infection, CCT3 mRNA expression in the BGC-823 and MGC-803 cells was reduced by 95% and 96%, respectively, as shown by quantitative RT-PCR. Western Blot showed significantly decreased CCT3 protein level in the shCCT3 lentivirus infected cells (bottom panels). **(B)** Growth curves of gastric cancer cell lines with or without CCT3 knockdown. Growth of BGC-823 and MGC-803 cells was inhibited with CCT3 Knockdown. **(C)** Cancer cell colony formation after CCT3 knockdown. Less colonies were formed from BGC-823 and MGC-803 cells infected with shCCT3-1 and shCCT3-2 lentivirus compared to cells infected with control lentivirus. (P<0.01).

To investigate the possible role of CCT3 in gastric cancer cell growth and survival, lentivirus expressing short hairpin RNA targeting CCT3 mRNA (shCCT3-1 and shCCT3-2) was used to infect human gastric cancer cell lines BGC-823 and MGC-803. Then the silencing effect of CCT3 in BGC-823 and MGC-803 cells was measured by qRT-PCR. After infection, the expression of CCT3 gene decreased by 94.6% and 95.7% in BGC-823 and MGC-803 cells compared with the shCtrl group, respectively. Knockdown of CCT3 protein expression in the gastric cancer lines was also confirmed by Western blotting. (Figure 2A). Silencing CCT3 also affected the proliferation of cell lines that was analyzed by MTT assays. With a dramatic decrease of CCT3 expression, the proliferation of the cancer cells in culture was significantly inhibited as shown by a nearly flat growth curve after lentiviral infection (Figure 2B). In addition, the colony-forming ability was clearly suppressed in shCCT3-1 and shCCT3-2 expressing cancer cells (Figure 2C), indicating a lower duplicating potential associated with CCT3 knockdown (P<0.01, *t*-test). In general, the results above suggested that expression of CCT3 is critical for gastric cancer cell proliferation.

### Knockdown of CCT3 expression promoted cancer cell apoptosis

**Figure 3:**
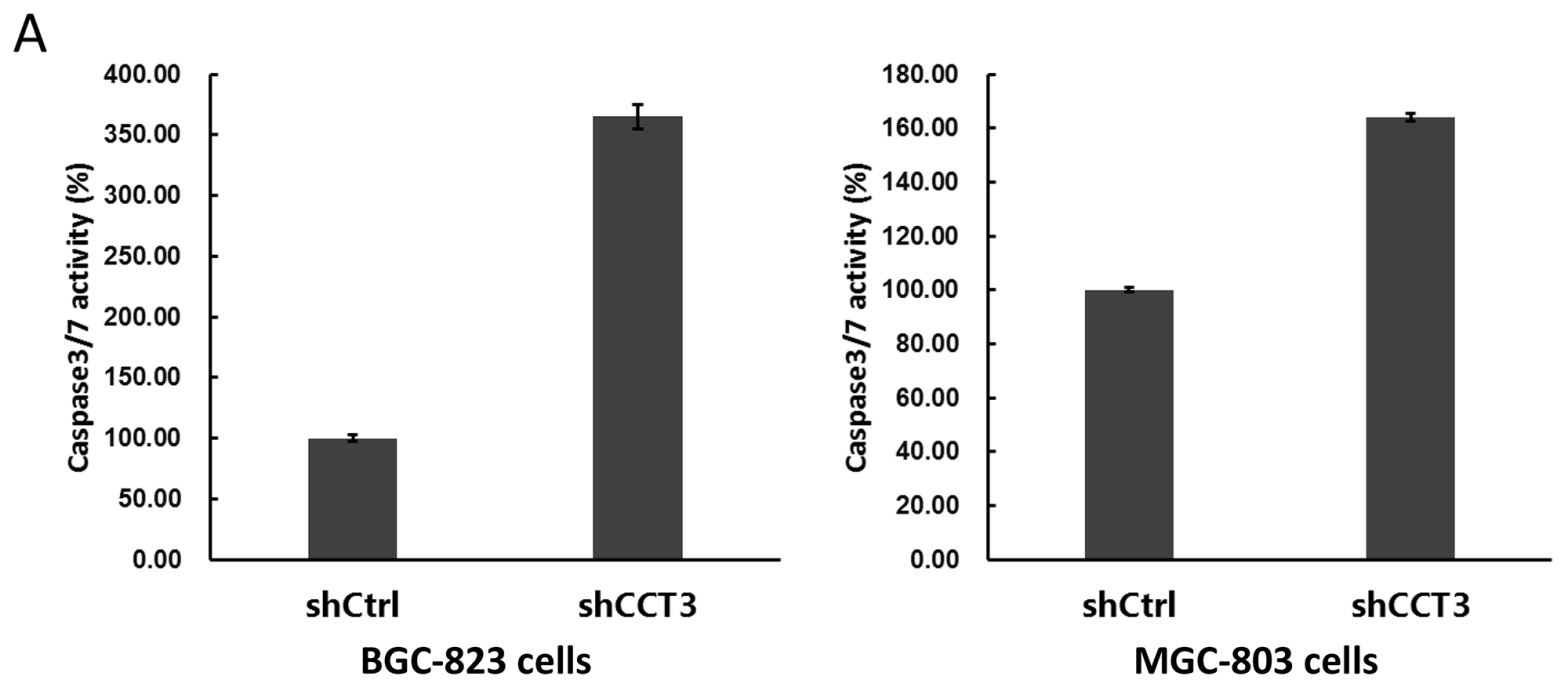

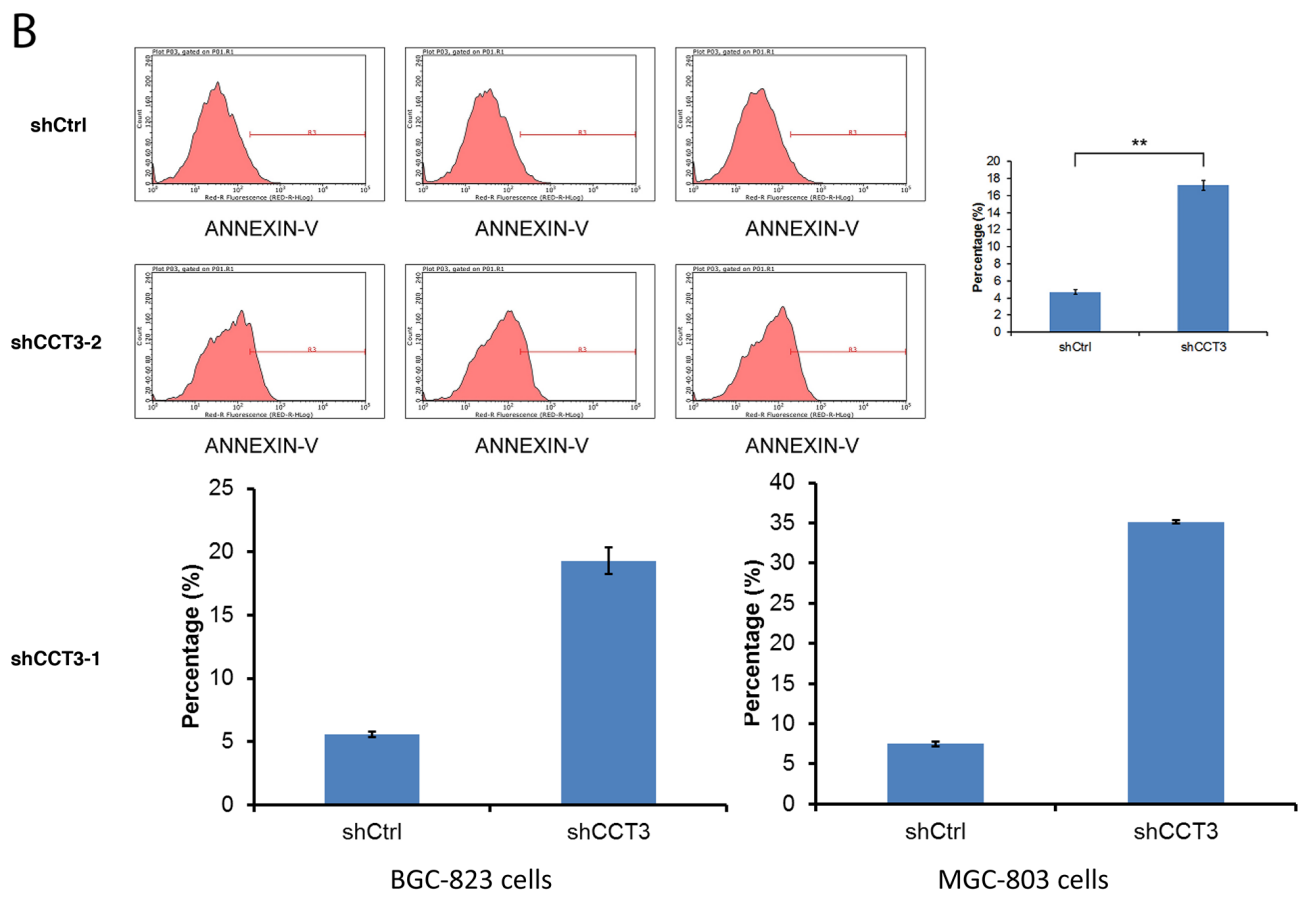

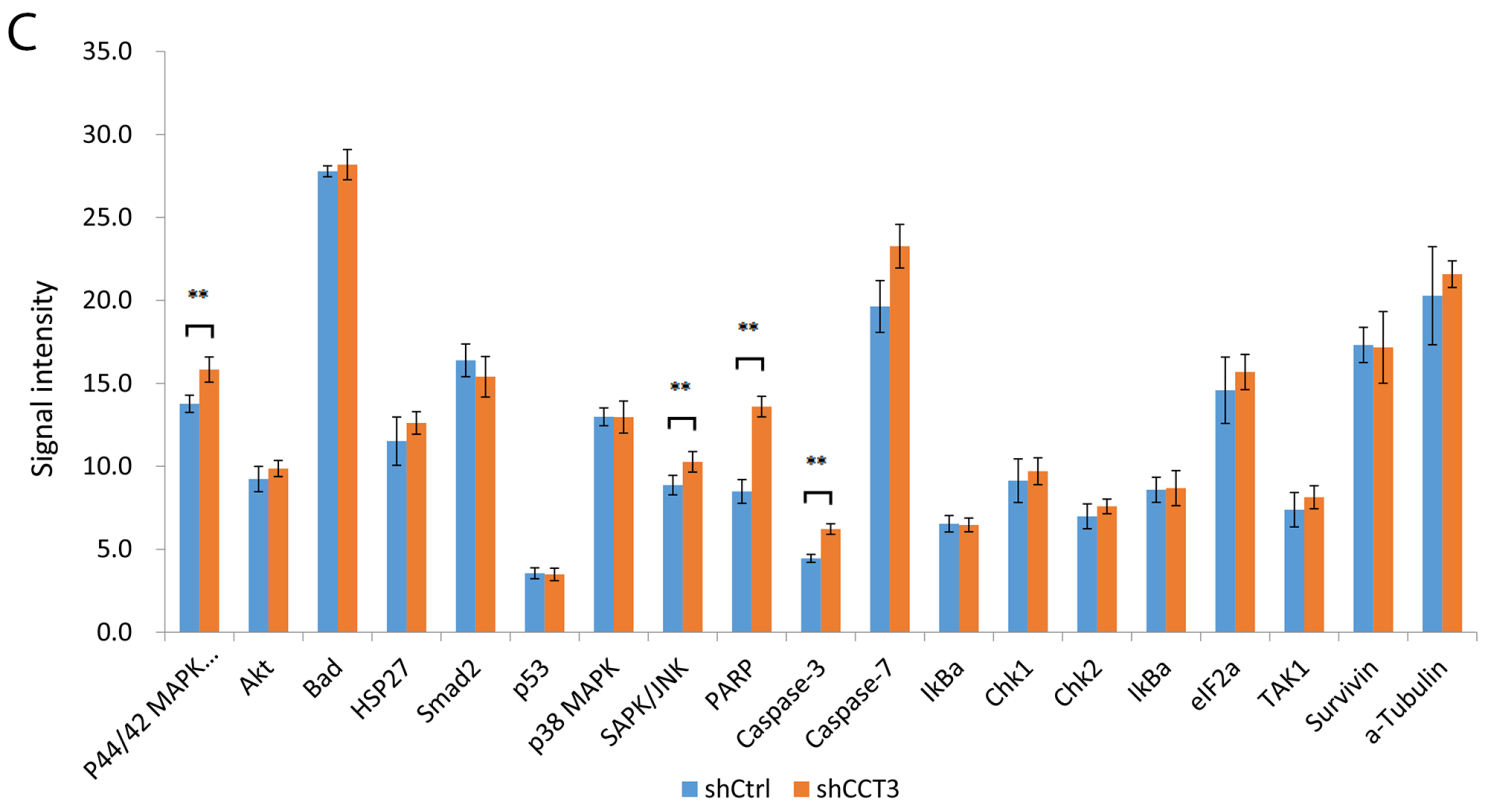
Knockdown of CCT3 promoted gastric cancer cell apoptosis **(A)** Activity of caspase 3/7 increased after knockdown of CCT3 in BGC-823 and MGC-803 cells. **(B)** Knockdown of CCT3 induced apoptosis in BGC-823 and MGC-803 cells. Increased number of apoptosis events were detected in shCCT3-1 and shCCT3-2 lentivirus infected gastric cancer cells, as shown by Annexin-V staining and FACS. **(C)** Antibody array detection of stress and apoptosis signaling molecules in BGC-823 cells with or without CCT3 knockdown. Increased expression of P44/42 MAPK, SAPK/JNK, PARP, Caspase-3 and -7 were shown in CCT3 knockdown cells. ^**^ p<0.05, *t*-test.

After shCCT3-1 and shCCT3-2 lentivirus infection, 19.3% of BGC-823 cells and 35.1% of MGC-803 cells underwent apoptosis. As a comparison, only 5.6% and 7.5% of shCtrl lentivirus infected cells underwent apoptosis, respectively (Figure 3B). The enhanced apoptotic event in CCT3 knockdown cells were also confirmed by caspase 3/7 activity assay, which showed a 3.6-fold and 1.6-fold increase in caspase 3/7 activity in the shCCT3 lentivirus infected BGC-823 and MGC-803 cells, respectively, compared to the shCtrl lentivirus infected cells (Figure 3A). These results suggested that decreased expression of CCT3 might increase apoptosis in gastric cancer cells.

### Knockdown of CCT3 expression inhibited cancer cell growth *in vivo*

**Figure 4:**
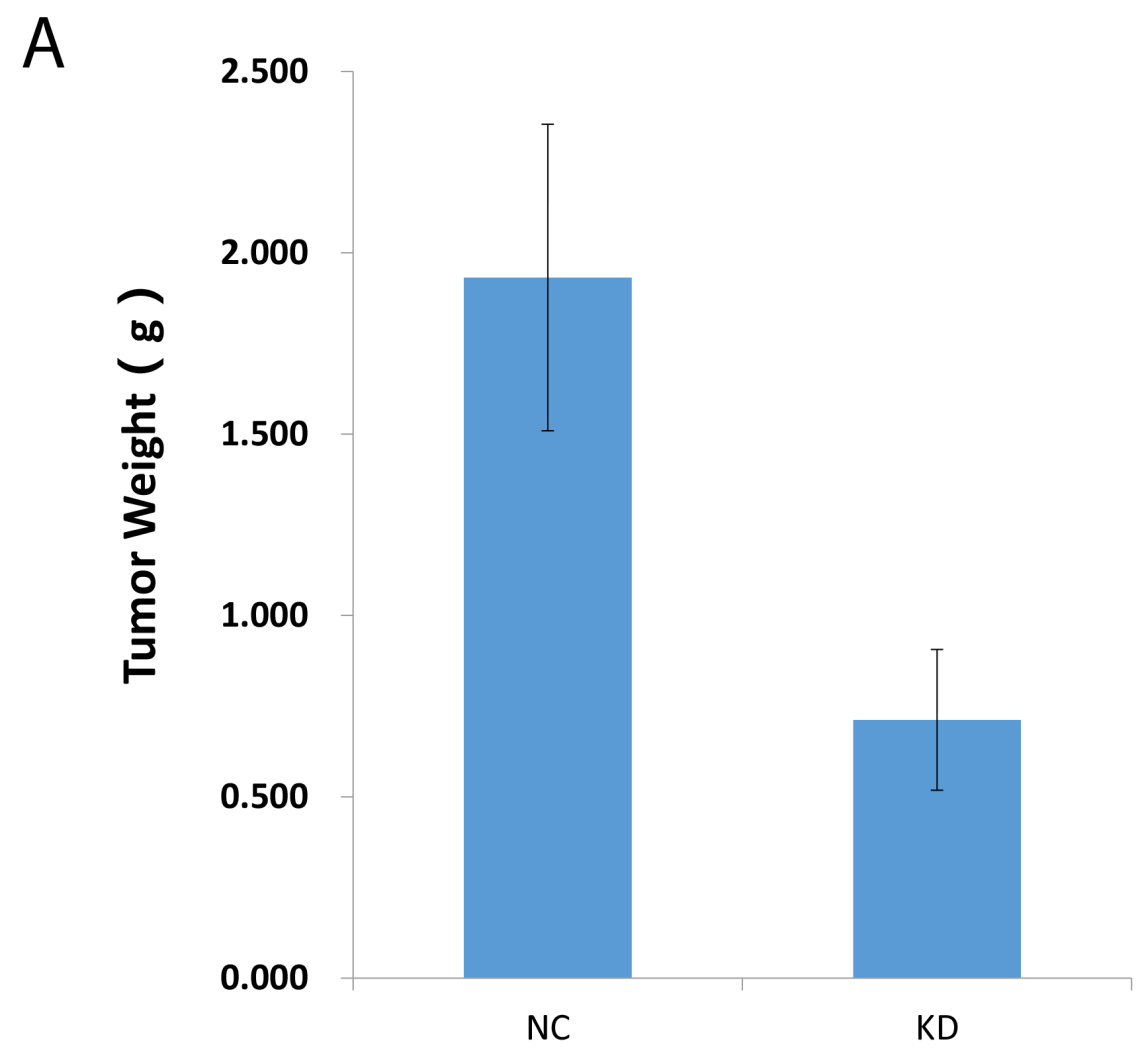

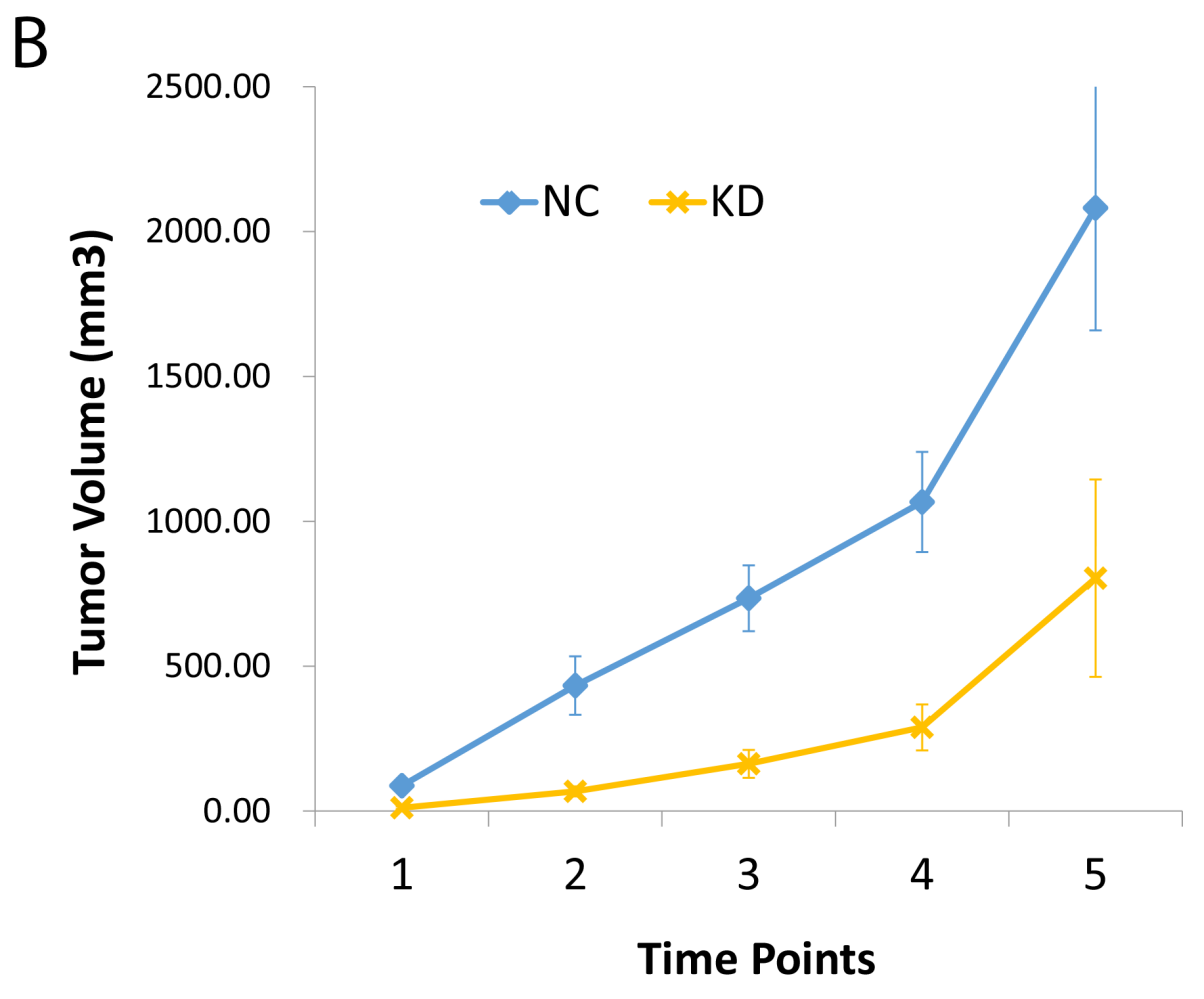
Knockdown of CCT3 suppressed gastric cancer cell growth in nude mice **(A)** 19 days after transplantation, xenografts from shCCT3-expressing BGC-823 cells grew smaller tumors by weight. **(B)** Sizes of tumors in nude mice after gastric cancer cell transplantation. Smaller tumors formed from transplanted BGC-823 cells with CCT3 knockdown (KD) compared to control BGC-823 cells (NC).

Now that a decreased CCT3 expression was shown to inhibit proliferation and promote apoptosis in gastric cancer cell lines *in vitro* (Figures 3A, 4B), it would be interesting to see if CCT3 knockdown confer any anti-tumor activity *in vivo*. To study the effects of CCT3 knockdown on xenograft tumor growth, BGC-823 cells infected with shCCT3-1 lentivirus or control virus were transplanted into nude mice. The growth of the cancer cells in the animals were monitored for 19 days. The results showed suppressed *in vivo* tumor growth with CCT3 knockdown (Figure 4A, 4B). Compared with NC group, the weight and volume of tumors in KD group were significantly reduced (P<0.05, *t*-test). The results suggested that CCT3 also act an important role in cell growth *in vivo*.

### Possible role of CCT3 in regulation of multiple signaling pathways in gastric cancer cells

To investigate the potential roles of CCT3 within the intricate networks of cellular pathways, a genome-wide differential study was performed on CCT3 knockdown MGC-803 cells and control cells using GeneChip PrimeView Human Gene Expression Array (Affymetrix, USA). The gene array analysis identified 859 upregulated and 887 downregulated genes in CCT3 knockdown cells (data not shown). The results were analyzed using Ingenuity^®^ Pathway Analysis (IPA^®^, Qiagen, USA) to identify possible relationships and pathways relevant to the gene expression profile. The bioinformatics analysis suggested that CCT3 may regulate the insulin-like growth factor-1 (IGF-1) signaling, actin cytoskeleton signaling and phosphatase and tensin homolog (PTEN) signaling pathways, which were known to play an important role in tumorigenesis of epithelia, cancer cell growth and survival.

**Figure 5:**
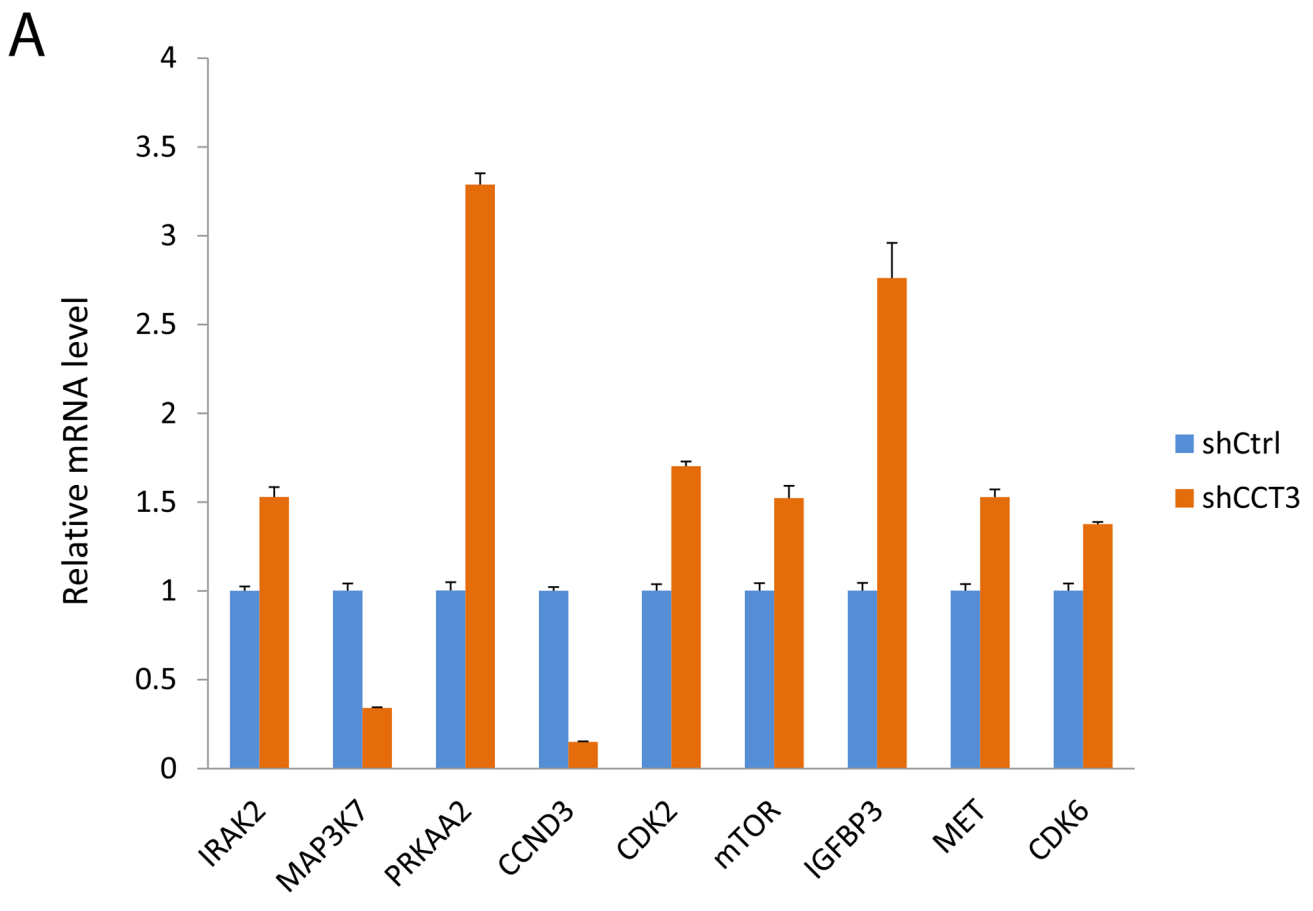

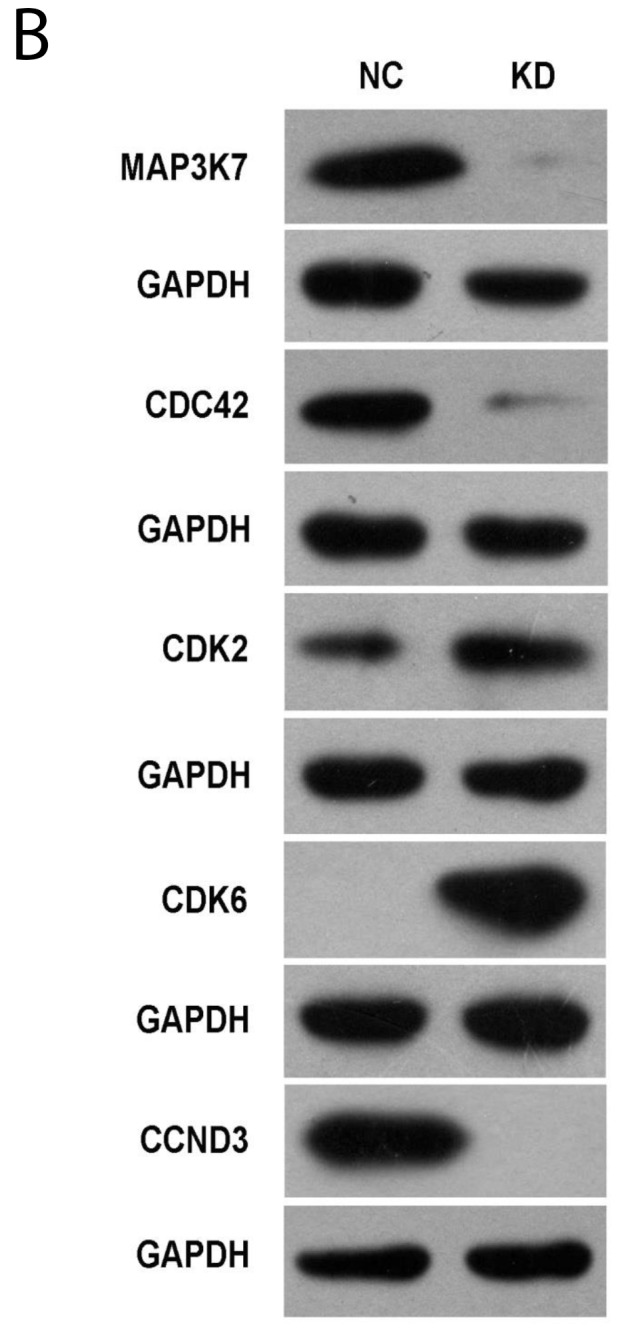
Gene expression affected by CCT3 knockdown **(A)** Expression of selected genes changed after knockdown of CCT3 in MGC-803 cells. qPCR showed upregulation of IRAK2, PRKAA2, CDK2, mTOR, IGFBP3, MET, CDK6 and downregulation of MAP3K7, CCND3 in CCT3 knockdown cancer cells. (*t* test, P<0.01 for all genes). **(B)** Western blotting showed increased levels of MAP3K7, CDC42, CCDN3 and decreased levels of CDK2 and CDK6 in the MGC-803 cells after CCT3 knockdown.

A penal of genes associated with these pathways was selected for further probe. Changes in gene expression after CCT3 knockdown were analyzed by quantitative RT-PCR. Significant changes were seen in interleukin 1 receptor associated kinase 2 (IRAK2), 5’-AMP-activated protein kinase catalytic subunit alpha-2 (PRKAA2), mechanistic target of rapamycin (mTOR), cyclin-dependent kinase 2 and 6 (CDK2, CDK6), Insulin-like growth factor binding protein 3 (IGFBP3), tyrosine-protein kinase MET, mitogen-activated protein kinase kinase kinase 7 (MAP3K7) and cyclin D3 (CCND3) (Figure 5A). Some of the gene expression changes shown by gene array were confirmed at protein level. For instance, Western blotting further confirmed the downregulation of MAP3K7, CCND3 and upregulation of CDK2, CDK6 showed that mitogen-activated protein kinase kinase kinase 7 (MAP3K7), cell division cycle 42 (CDC42) and cyclin D3 (CCND3) were downregulated in CCT3 knockdown cells (Figure 5B), while cyclin-dependent kinase 2 and 6 (CDK2, CDK6) were upregulated in CCT3 knockdown cells (Figure 5B). These genes are known to involve in cancer cell proliferation, stress response, apoptosis and growth regulation, although their exact roles in the development of gastric cancer have yet to be determined. Our current study in CCT3 and associated pathways might provide information useful for future investigation of molecular mechanism of gastric cancer.

## DISCUSSION

Group II chaperonin CCT has been shown to modulate the folding of cellular proteins involved in oncogenesis, such as VHL, p53, STAT3 and cell cycle regulator CDC20. Recently, CCT/TRiC was also demonstrated to facilitate the folding of AML1-ETO, an oncofusion protein responsible for the development of acute myeloid leukemia. With a large number of substrates representing a broad spectrum of functionalities, CCT is probably involved in many other oncogenesis pathways. It is not surprising that CCT or its subunits have been implicated in the development of various types of cancers, such as breast [15, 22], liver [17, 20, 23], colon [24, 25], lung cancer [26] and glioma [27]. However, little is known regarding the role of CCT3 in the development of gastric cancer. In this report, we demonstrated that CCT3 was upregulated in the primary gastric cancer tissues and knockdown of CCT3 expression in the gastric cancer cell lines suppresses cancer cell proliferation and promote apoptosis. These results were similar to those observed in CCT1 and CCT8 knockdown experiments [23, 28], suggesting a common effect of reduced TRiC assembly due to knockdown of CCT subunits.

The CCT subunits may function as part of the chaperonin complex to orchestrate protein folding. However, the precise mechanism of action of the system remains to be elucidated. The CCT subunits have greatly diverged substrate-binding domains, which are predicted to allow for specific substrate interaction [29]. It is possible that deficit in one CCT subunits might affect certain substrates more readily than others do. In the study reported by Huang et al., knockdown of CCT8 dramatically reduced the level of CDK2 in hepatocellular carcinoma cells [23]. In our study, knockdown of CCT3 increased CDK2 and CDK6 level. The reason behind this discrepancy is unknown. CDK6-cyclin D is responsible for limited phosphorylation of the retinoblastoma tumor suppressor (Rb) protein. And activation of CDK2-cyclin E leads to further phosphorylation and inactivation of pRb, release of E2F, and full commitment to S-phase entry [32]. It could be that folding of CDK2 depends on specific interaction with CCT8 but not CCT3. The other possibility could be that CCT or its subunits might play different roles in different cancer cells.

Moreover, it was postulated that individual CCT subunits might also function independently without forming the hetero-oligomeric complex [30]. For instance, CCT4 and CCT5 homo-oligomers have been found to form 8-fold double rings absent the other subunits. And the CCT4 or CCT5 oligomer rings exhibited activities of ATP hydrolysis and protein folding comparable to the TRiC ring [31]. Whether CCT3 have any functional autonomy is currently unknown. It is possible that CCT3 might play a more specific role in gastric cancer cells other than functioning as a component of TRiC. Our study has demonstrated a critical role of CCT3 in the growth and survival of gastric cancer cells. Further studies to determine specific substrates for CCT3 and the precise function of CCT3 in gastric cancer will help development of new cancer therapies targeting CCT3.

## MATERIALS AND METHODS

### Tumor tissues and immunohistochemistry

Informed consent was obtained from 26 patients who underwent gastric cancer resection. The paraffin-embedded tissue samples of primary tumors and adjacent non-cancerous tissues from these patients were stained for CCT3 with rabbit CCT3 antibody (1:50, Abcam, ab1774255) using standard Immunohistochemistry method. The intensity of the stain in cytoplasm was score as follows: “negative”=0; “weak”=1; “moderate”=2 and “strong”=3. The percentage of positively stained cells in a section was scored as 0%=0, 1-25%=1; 26-50%=2; 51-75%=3 and 76-100%=4. Only cancer cells and epithelial cells were evaluated for staining. The immunostaining score of a tissue section was expressed as the product of its intensity score and positive percentage score. An immunostaining score ≤6 was arbitrarily classified as low CCT3 expression and a score >6 high CCT3 expression. Four (4) sections of primary tumor and Four (4) sections of adjacent epithelia from each subject were evaluated. Totally 104 primary cancer sections and 104 non-cancerous sections were scored and assigned to high or low CCT3 expression group. Significant level was tested with Fisher’s exact test.

### CCT3 shRNA, lentiviral vector and virus production

Two short hairpin RNA (shRNA), shCCT3-1 and shCCT3-2 were designed based on the target sequences 5’CAAGTCCATGATCGAAATT3’ and 5’ GCAAGGCATTGGATGATAT 3’ on CCT3 mRNA respectively. A DNA oligonucleotide containing the target hairpin structure, transcription termination sequence and proper restriction sites was synthesized and inserted into the multiple cloning site of lentiviral vector GV115 (GeneChem, Shanghai, China). A control vector containing hairpin sequence unrelated to the target was constructed using same technique. Lentiviral shRNA constructs were confirmed by sequencing and were used for lentivirus production. Virus packaging and purification were performed by GeneChem (Shanghai, China) using the company’s lentiviral expression system.

### Cell culture and lentivirus infection

Human gastric cancer cell line MGC-803 and BGC-823 were maintained in RPMI-1640 medium containing 10% fetal bovine serum at 37°C with 5% CO_2_. Medium was changed every 3 days. Lentivirus infection was performed on cells at 80% confluency, with a multiplicity of infection (MOI) of 50. 72 hours after infection, the cells were used for downstream assay or transplantation.

### Quantitative RT-PCR

Total RNA was extracted from lentivirus infected gastric cancer cells using Trizol. cDNA synthesis was performed using M-MLV Reverse Transcriptase (M1705, Promega, USA) following manufacturer’s instruction. Real-time PCR was performed with 7500 Real Time PCR system (Applied Agilent, USA) using SYBR Master Mixture (DRR041B, Takara, Otsu, Japan). Relative expression of CCT3 was calculated as 2^-ΔΔCt^ using GAPDH as internal reference. The sequences of the primer pairs were: CCT3 forward: 5’-TCAGTCGGTGGTCATCTTTGG-3’; CCT3 reverse: 5’- CCTCCAGGTATCTTTTCCACTCT-3’; GAPDH: 5’-TGACTTCAACAGCGACACCCA-3’; GAPDH reverse: 5’-CACCCTGTTGCTGTAGCCAAA-3’. Sequences of the other primers used in this study are provided in the Supplement of this article. Relative expression of genes was calculated as 2-ΔΔCt using GAPDH as internal reference. Reactions were performed in triplicate. Statistical significance was tested using *t*-test.

### Cell proliferation analysis (MTT assay)

After lentivirus infection, gastric cancer cell line MGC-803 and BGC-823 were plated on 96-well plates (2000 cells/well in triplicate). Five plates were prepared at the same time and each plate was tested at one of the time points: 24 hours, 48 hours, 72 hours, 96 hours and 120 hours. MTT (3-(4, 5-dimethylthiazolyl-2)-2, 5-diphenyltetrazolium bromide) was purchased from Genview (Houston TX, USA; JT343). MTT assay was performed following manufacturer’s instruction. The absorbance at 490nm (OD490) was then used as surrogate for cell number and plotted on the cell growth curve. Statistical significance was tested using *t*-test.

### Tumor colony-forming assay

Tumor colony-forming assay was performed as previously described [21]. In brief, three days after lentivirus infection, BGC-823 and MGC-803 cells were plated on 6-well plate (300 cells/well and 400 cells/well, respectively). BGC-823 cells were incubated in growth medium for 8 days and MGC-803 cells for 10 days to allow colony formation. Tumor cell colonies were counted after fixation and Giemsa stain. Statistical significance was tested using *t*-test.

### Apoptosis assays

Caspase 3/7 activity of the cancer cells after lentivirus infection was assessed with Caspase-Glo® 3/7 Assay Systems (G8091, Promega, USA) following the instructions of product manual.

Early apoptosis events were detected with Annexin-V APC detection kit (Cat. 88-8007, eBioscience, USA) following the instructions of product manual. Fluorescence-activated cell sorting was performed using Guava easyCyte HT cytometry system (Millipore, USA). Statistical significance was tested using *t*-test.

### Tumor cell transplantation

Lentivirus infected BGC-823 cells were selected with growth media containing 4μg/ml of Puromycin (Cat. 631305, Clontech, USA) for 48 hours. After selection, cells (4.0X106 cells per mouse) were harvested and injected subcutaneously to the right axilla of Balb/c Nude mice (female, 4 weeks old). The length (L) and width (W) of the tumor were measured at day 7, 11, 14, 17 and day 19 after transplantation. Tumor size was calculated as π/6×L×W2 (mm3). Animals were sacrificed at day 19 and the tumors were resected and weighed. Statistical significance was tested using *t*-test.

### Western blotting

Western blotting was carried out on lysates of gastric cancer cell line MGC-803 as described previously [21] using mouse anti-GAPDH (1:4000, Santa Cruz, SC-32233), Anti-CCT3 (1:200, Abcam, ab174255) rabbit anti-MAP3K7 (1:1000, Abcam, ab109526), rabbit anti-CDC42 (1:1000, Abcam, ab187643), rabbit anti-CDK2 antibodies (1:1000, CST, #2546), mouse anti-CDK6 (1:1000, CST, #3136) and mouse anti-CCND3 (1:1000, CST, #2936). The secondary antibody was an HRP-conjugated anti-rabbit IgG antibody (1:5000, Santa Cruz, sc-2004) or anti-mouse IgG (1:5000, Santa Cruz, sc-2005). The enhanced chemiluminescence reagent (Cat. 32106, ThermoFisher, USA) was used for signal detection.
